# Laparoscopic Management of Gynaecological Emergencies: A Single-Centre Prospective Observational Study

**DOI:** 10.7759/cureus.112884

**Published:** 2026-07-17

**Authors:** Azhar Un Nisa Quraishi, Kavitha Yogini Duraisamy, Syed Aadil S Andrabi, Vishnu Priya G, Suganya Ganapathirajan, Palanivelu Chinnusamy

**Affiliations:** 1 Department of Endogynecology, GEM Hospital and Research Centre, Coimbatore, IND; 2 Department of Urology, Sher-i-Kashmir Institute of Medical Sciences, Srinagar, IND; 3 Department of Surgery, PSG Institute of Medical Sciences & Research, Coimbatore, IND; 4 Department of Surgical Gastroenterology, GEM Hospital and Research Centre, Coimbatore, IND

**Keywords:** adnexal torsion, ectopic pregnancy, gynecological emergencies, laparoscopy, pelvic inflammatory disease

## Abstract

Background: Gynecological emergencies require rapid diagnosis and timely intervention to prevent morbidity and preserve fertility. Laparoscopy has increasingly replaced laparotomy in stable patients due to its minimally invasive advantages.

Methods: This prospective observational study was conducted at GEM Hospital, Coimbatore (June 2024-June 2025), including 46 hemodynamically stable women with acute gynecological emergencies. Clinical, imaging, intraoperative, and postoperative data were recorded and analyzed descriptively.

Results: The mean age was 27.9 ± 11.1 years. Acute abdominal pain was the most common presentation (95.7%). Ultrasound confirmed pathology in 95.7% of cases. Adnexal torsion was the most frequent diagnosis (60.8%), followed by ectopic pregnancy (23.9%) and pelvic inflammatory disease (6.5%). Torsion was mainly managed with detorsion, cystectomy, and oophoropexy, achieving high ovarian preservation. Ectopic pregnancies were primarily treated with salpingectomy. All cases were completed laparoscopically without conversion. The operative time ranged from 50 to 160 minutes. Postoperative recovery was favorable, with 87% having no complications; minor complications occurred in 13%, with no major morbidity or readmissions.

Conclusion: Laparoscopy is a safe and effective approach for gynecological emergencies in stable patients, offering accurate diagnosis, organ preservation, and rapid recovery.

## Introduction

Gynecological emergencies comprise a broad array of acute presentations that demand rapid diagnosis and timely intervention to avert significant morbidity, preserve future fertility, and, in extreme cases, prevent mortality. Among these, ectopic pregnancy, adnexal torsion, ruptured ovarian cysts, acute pelvic inflammatory disease (PID), and hemorrhagic corpus luteum cysts represent the most common entities encountered in tertiary care settings [1]. In our department at GEM Hospital Coimbatore, these conditions account for nearly 15% of all admissions, reflecting both the regional disease burden and patterns of healthcare access.

Ectopic pregnancy - implantation of the fertilized ovum outside the uterine cavity - remains a critical gynecological emergency. Its incidence has risen alongside assisted reproductive techniques and pelvic inflammatory risk factors, now affecting approximately 1-2% of all pregnancies [2,3]. Clinically, patients often present with abdominal pain, vaginal bleeding, and hemodynamic instability. Early detection via transvaginal ultrasonography and serial β-hCG assays has shifted management from emergent laparotomy to more nuanced, minimally invasive approaches when appropriate [4,5].

Adnexal torsion, defined as twisting of the ovarian pedicle on its vascular axis, leads to venous congestion, arterial compromise, and potential loss of ovarian tissue. Although it comprises a smaller percentage of gynecological emergencies, prompt intervention is critical to preserve endocrine and reproductive function, especially in young women [6,7]. Preoperative diagnosis remains challenging; while Doppler ultrasound can suggest absent blood flow, it lacks sensitivity, and clinical suspicion often mandates diagnostic laparoscopy to confirm and treat the torsion [8].

Ruptured ovarian cysts - whether functional or pathological - may cause intra-abdominal bleeding, peritonitis, or hemoperitoneum. While many resolve spontaneously, those complicated by hemodynamic compromise or persistent pain are best managed surgically. In such cases, laparoscopic evaluation allows for direct visualization of the rupture site, control of bleeding, and cyst excision if indicated [9].

Acute PID, when complicated by tubo-ovarian abscess or peritonitis, presents with fever, pelvic pain, and elevated inflammatory markers. Although antibiotic therapy is first-line, surgical drainage is frequently required to prevent chronic sequelae, including infertility and chronic pelvic pain [10]. Laparoscopic drainage and adhesiolysis reduce adhesion formation compared to open surgery and enable targeted irrigation of infected spaces under direct vision [11,12].

In India, data regarding emergency laparoscopy remain limited. Single-centre audits from tertiary hospitals report growing uptake but underscore challenges-such as resource constraints, uneven training, and patient delays-that impact clinical outcomes. At GEM Hospital Coimbatore, our department aims to standardize minimally invasive care for these critical emergencies.

## Materials and methods

Study design and setting

This prospective observational case series was conducted in the Department of Endogynecology, GEM Hospital, Coimbatore, between June 1, 2024, and June 30, 2025. The study was approved by the Institutional Ethics Committee (dated 06/09/2025), and written informed consent was obtained from all participants prior to enrollment.

Women presenting to the emergency gynecology service with acute pelvic pain and suspected gynecological emergencies were screened for inclusion in the study. Patients with a clinical or radiological suspicion of adnexal torsion, ectopic pregnancy, ruptured ovarian cyst, hemorrhagic corpus luteum cyst, or complicated pelvic inflammatory disease were considered eligible. Following initial assessment and resuscitation, only hemodynamically stable patients who were suitable candidates for laparoscopic management were included. Patients with uncorrected coagulopathy, contraindications to general anesthesia, suspected gynecological malignancy on preoperative imaging, or persistent hemodynamic instability requiring immediate laparotomy were excluded from the study.

All patients underwent detailed history taking and clinical examination at presentation. Baseline demographic characteristics, parity, presenting symptoms, duration of symptoms, and physical examination findings were recorded. Laboratory investigations included complete blood count, blood grouping and cross-matching when indicated, serum beta-human chorionic gonadotropin testing in suspected ectopic pregnancy, and other investigations as clinically required. Radiological assessment was performed using transabdominal and/or transvaginal ultrasonography in all patients. Magnetic resonance imaging was selectively utilized in patients with indeterminate ultrasonographic findings or when further characterization of adnexal pathology was required.

After preoperative evaluation and optimization, patients underwent emergency laparoscopy under general anesthesia. Intraoperative findings were documented, including the underlying pathology, laterality of disease, adnexal viability, presence of hemoperitoneum, adhesions, tubo-ovarian abscess, endometriosis, or other associated pelvic pathology. Surgical management was individualized according to the intraoperative findings and underlying diagnosis. In patients with adnexal torsion, conservative procedures such as detorsion, cystectomy, and oophoropexy were performed whenever ovarian preservation was considered feasible. Salpingectomy or tubal milking was performed for ectopic pregnancy as appropriate. Patients with pelvic inflammatory disease and tubo-ovarian abscess underwent drainage, adhesiolysis, salpingectomy, partial oophorectomy, or definitive surgery based on disease extent and reproductive considerations. Endometriotic cyst rupture and corpus luteal cyst rupture were managed laparoscopically with cystectomy, deroofing, hemostasis, and ovarian reconstruction where indicated.

Operative duration, intraoperative diagnosis, procedure performed, requirement for blood transfusion, conversion to laparotomy, and perioperative complications were prospectively recorded. Postoperatively, patients were monitored for fever, ileus, hemorrhage, need for high-dependency unit admission, and other complications. Length of hospital stay and need for readmission or reoperation were also documented.

Statistical analysis

All data from the 46 patients were recorded in a prospectively maintained master chart and analyzed using SPSS Statistics for Windows, version 26.0 (IBM Corp., Armonk, NY, USA). Continuous variables are presented as mean ± standard deviation (SD), median with interquartile range (IQR), and range, as appropriate, while categorical variables are shown as counts and percentages.

As this was a descriptive observational study, no formal statistical comparisons between subgroups were undertaken. The findings are therefore reported as descriptive summaries, intended to clearly convey operative trends, perioperative outcomes, and complication patterns across the cohort.

## Results

**Figure 1:**
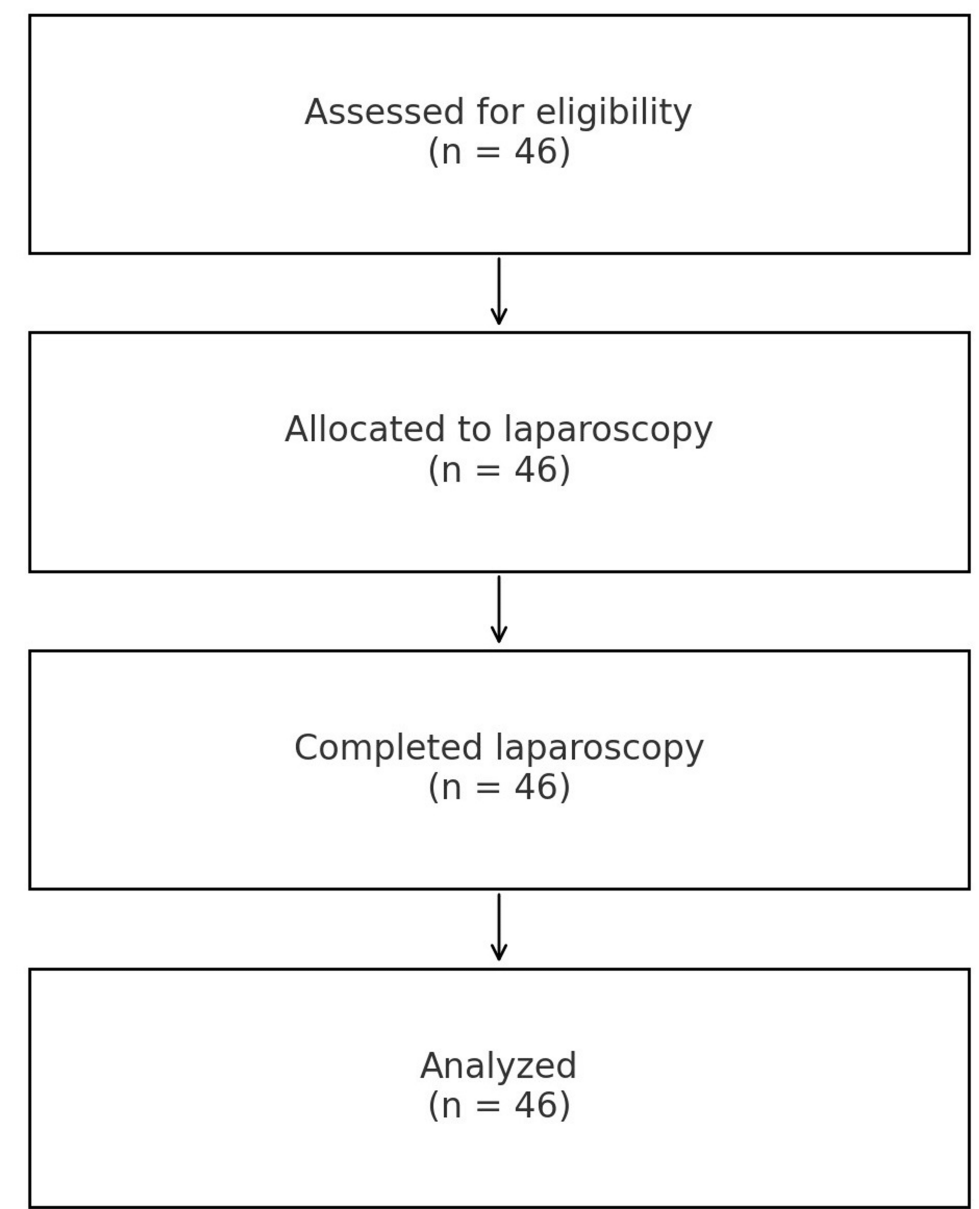
Flow diagram of patient enrollment and analysis (n = 46).

Between June 1, 2024 and June 30, 2025, 46 consecutive women presenting with suspected gynecological emergencies at the Endogynecology Unit of GEM Hospital, Coimbatore, were enrolled in this observational study. All patients met the inclusion criteria (i.e., acute pelvic pain or hemodynamic signs of intra-abdominal pathology) and provided informed consent. There were no exclusions, and all 46 cases proceeded to planned operative laparoscopy. No conversions to open surgery occurred, and all 46 cases were included in the final analysis (Figure 1).

In this prospective cohort of 46 women, the age profile was clustered in the reproductive years, with a mean of 27.9 ± 11.1 years (range 11-61 years). Parity was skewed toward nulliparity: 25 patients (54%) had no prior births, 12 (26%) were primiparous, six (13%) had two deliveries, and three (7%) had three or more. Clinically, 44 women (96%) presented with sudden-onset lower abdominal pain, often severe enough to require emergency admission; nausea and vomiting accompanied pain in 16 cases (35%), while fever (two; 4%) and vaginal bleeding (one; 2%) were uncommon. The interval from symptom onset to surgery ranged from 0.5 to 14 days (median four days, IQR 1-7). On examination, adnexal tenderness was nearly universal (43/46; 93.5%), frequently with guarding. Preoperative imaging proved indispensable: transvaginal or transabdominal ultrasound confirmed adnexal lesions in 44 women (95.7%) and raised suspicion for torsion in 18 cases (39.1%), while pelvic MRI, performed in 18 patients (39.1%) with complex or indeterminate ultrasound findings, offered superior soft-tissue contrast to characterize hemorrhagic, endometriotic, or inflammatory changes (Table 1).

**Table 1:** Baseline characteristics, clinical presentation, and imaging findings

Variable	Category	Value
Age (years)	Mean ± SD	27.9 ± 11.1
	Median (IQR)	27.0 (19.2–31.8)
	Range	11–61
Parity	0	25 (54.3%)
	1	12 (26.1%)
	2	6 (13%)
	≥3	3 (6.5%)
Symptoms	Abdominal pain	44 (95.7%)
	Nausea and vomiting	16 (34.8%)
	Fever	2 (4.3%)
	Vaginal bleeding	1 (2.2%)
Imaging	Ultrasound	44 (95.7%)
	Ultrasound suspicious of torsion	18 (39.1%)
	MRI	18 (39.1%) – characterization of hemorrhagic/endometriotic/inflammatory lesions

In this cohort of 46 women, adnexal torsion was the most frequent emergency, occurring in 28 patients (60.8%). Tubal ectopic pregnancy followed with 11 cases (23.9%), acute PID (pyosalpinx/tubo-ovarian abscess) in three (6.5%), endometriotic cyst rupture in two (4.3%), corpus luteal cyst rupture in one (2.2%), and uterine torsion in one patient (2.2%) (Table 2).

**Table 2:** Underlying pathologies PID: pelvic inflammatory disease

Underlying pathology	Number of patients	Percentage (%)
Adnexal torsion	28	60.8
Tubal ectopic pregnancy	11	23.9
Acute PID (pyosalpinx/tubo-ovarian abscess)	3	6.5
Endometriotic cyst rupture	2	4.3
Corpus luteal cyst rupture	1	2.2
Uterine torsion	1	2.2

Management was tailored to preserve ovarian function and address the underlying lesion. Of the 28 cases of adnexal torsion, 22 women (47.8% of the cohort; 78.6% of torsion cases) underwent detorsion followed by cystectomy with oophoropexy to detorse and secure the adnexa. One (2.2% of the cohort; 3.6% of torsion cases) patient had ovarian torsion alone, which was managed by oophoropexy only. Five patients (10.9% of the cohort; 17.8% of the torsion cases) with torsion underwent detorsion, followed by unilateral salpingo-oophorectomy for nonviable ovarian tissue. Opposite-sided oophoropexy was done in such cases. Salpingectomy was performed in 10 ectopic pregnancies (21.7%), with one case (2.2%) managed by careful tubal milking. Milking was done as the patient’s opposite tube was already removed due to a previous history of ruptured tubal ectopic pregnancy. Among three PID cases, one (2.2%) was managed with abscess drainage, bilateral pyosalpinx excision, and complete salpingectomy. The other one (2.2%) needed additional bilateral partial oophorectomy due to bilateral tubo-ovarian abscess demonstrated intraoperatively. One pyosalpinx (2.2%) required total laparoscopic hysterectomy with bilateral salpingo-oophorectomy. The patient had an enlarged uterus (20 weeks) with multiple fibroids and was keen for definitive management. Two ruptured endometriotic cysts (4.3%) underwent deroofing and ovarian reconstruction. One case of uterine torsion (2.2%) due to a huge ovarian cyst also underwent total laparoscopic hysterectomy with bilateral salpingo-oophorectomy due to the postmenopausal state. One (2.2%) case of corpus luteal cyst rupture was managed by cystectomy with bilateral oophoropexy. All 46 procedures were completed laparoscopically without conversion to laparotomy (Table 3).

**Table 3:** Procedures performed U/L: unilateral, B/L: bilateral, TLH: total laparoscopic hysterectomy, BSO: bilateral salpingo-oophorectomy

Underlying pathology		Procedure done	Number of patients	Percentage
Torsion	Adnexal torsion	Cystectomy with oophoropexy	22	47.8
		Oophoropexy alone	1	2.2
		U/L salpingo-oophorectomy with C/L oophoropexy	5	10.9
	Uterine torsion	TLH with BSO	1	2.2
Ectopic pregnancy		Salpingectomy	10	21.7
		Tubal milking	1	2.2
Acute PID-pyosalpinx		B/L pyosalpinx excision with salpingectomy	1	2.2
		B/L pyosalpinx excision with salpingectomy with B/L partial oophorectomy	1	2.2
		TLH with BSO	1	2.2
Ruptured endometriotic cyst		Deroofing with ovarian reconstruction	2	4.3
Ruptured Corpus luteal cyst		Cystectomy with B/L oophoropexy	1	2.2

Operative duration varied in accordance with procedural complexity. The most expedient intervention was oophoropexy for ovarian torsion, which averaged 50 minutes (range 40-65 minutes). Cystectomy with oophoropexy - our most frequent operation - required a mean of 60 minutes (45-75 minutes), reflecting straightforward detorsion and adnexal anchoring. Salpingectomy for ectopic pregnancy averaged 70 minutes (50-90 minutes), and management of tubo-ovarian abscess with adhesiolysis and partial oophorectomy required 120 minutes (100-150  minutes). Endometriotic cyst deroofing and reconstruction was completed in 80 minutes (60- 100 minutes). One case of endometriotic cyst rupture was complicated by intestinal obstruction, and multiple bowel loops were adherent to the anterior abdominal wall. Moreover, endometriotic cysts were densely adherent to the rectosigmoid and posterior surface of the uterus. Adhesiolysis was done, and hence the procedure was prolonged with a mean duration of 160 minutes. Total laparoscopic hysterectomy with bilateral salpingo-oophorectomy and uterosacral colpopexy had a mean duration of 120 minutes (100-150 minutes) (Table 4).

**Table 4:** Operative time by procedure

Procedure	Mean operative time (min)	Range (min)
Oophoropexy for ovarian torsion	50	40–65
Cystectomy with oophoropexy	60	45–75
Salpingectomy for ectopic pregnancy	70	50–90
Total laparoscopic hysterectomy with bilateral salpingo-oophorectomy	120	100-150
Adhesiolysis, deroofing, and reconstruction	160	150-170
Deroofing of endometriotic cyst and reconstruction	80	60-100

Early postoperative recovery was excellent in the majority of our patients (40/46; 87.0%). Four women (8.7%) developed low-grade fever on postoperative days 1-2, which responded promptly to antipyretics and did not require prolongation of their hospital stay. Blood transfusion was required in three patients. Two patients needed observation in the High-Dependency Unit (HDU) for 24 hours. One patient had postoperative ileus. No patient required readmission, relaparoscopy, or experienced major morbidity, such as deep pelvic abscess or significant hemorrhage. These findings underscore the safety profile of emergency laparoscopy in our hands, with minimal impact on overall recovery and excellent early outcomes (Table 5).

**Table 5:** Postoperative outcomes

Postoperative outcome	Number of patients	Percentage (%)
No postoperative complication	40	87
Low-grade fever	4	8.7
Blood transfusion	3	6.5
HDU observation	2	4.3
Postoperative ileus	1	2.2
Readmission	0	0
Re-laparoscopy	0	0
Major morbidity	0	0

## Discussion

In our observational study of 46 women managed with a laparoscopy-first protocol for gynaecological emergencies, the mean age was 27.9 ± 11.1 years (range 11-61), and 54.3% were nulliparous - demographics consistent with cohorts dominated by reproductive-age patients in whom fertility preservation guides management, as reported by Oelsner et al. [13] and emphasized by Vural and Şik [14]. Acute abdominal pain was the principal presentation (95.7%), with nausea/vomiting (34.8%), fever (4.3%), and vaginal bleeding (2.2%) occurring less frequently; this distribution mirrors patterns in torsion and ruptured-cyst series by Oelsner and Shashar [6], Teng et al. [9], and Pansky et al. [15]. Ultrasonography confirmed adnexal pathology in 95.7% and suggested torsion in 39.1%, aligning with the recognised limitations of Doppler for torsion diagnosis described by Chang et al. [16]. Selective MRI use (39.1%) for complex or equivocal cases accords with problem-solving recommendations in the Royal College of Obstetricians and Gynaecologists (RCOG) Green-top Guideline No. 21 [17].

The intraoperative spectrum was led by adnexal torsion (28/46; 60.8%), followed by tubal ectopic pregnancy (11/46; 23.9%), acute pelvic inflammatory disease with tubo-ovarian abscess (3/46; 6.5%), ruptured endometriotic cyst (2/46; 4.3%), corpus luteal cyst rupture (1/46; 2.2%), and uterine torsion (1/46; 2.2%). This distribution is concordant with institutional audits and pathology-specific series in which torsion and ectopic pregnancy predominate among emergency laparoscopic indications (Kruszka and Kruszka [1]; Alkatout et al. [3]; Huchon and Fauconnier [7]). Within the torsion subgroup, detorsion with cystectomy and oophoropexy was undertaken in 22/28 (78.6%; 47.8% of the cohort), oophoropexy alone in 1/28 (3.6%), and unilateral salpingo-oophorectomy in 5/28 (17.8%; 10.9% of the cohort) for non-viable adnexa, yielding an ovarian preservation rate of 82.1% (23/28). This high preservation range is consistent with the maximal salvage rates reported in minimal surgery approaches by Oelsner et al. [13]. The routine use of fixation alongside detorsion in our series aligns with Pansky et al. [15], who demonstrated a significant reduction in retorsion when oophoropexy accompanies conservative management.

Ectopic pregnancy constituted 11/46 (23.9%) cases; operative management was predominantly salpingectomy (10/11; 90.9%), with tubal milking in 1/11 (9.1%). The mean operative time for salpingectomy was 70 minutes (50-90), which is comparable to established benchmarks for minimally invasive ectopic management. These findings are consistent with the high procedural reliability and effectiveness of laparoscopic surgery synthesized in the Cochrane review by Hajenius et al. [4] and the fertility-preserving perspective outlined by Alkatout et al. [3].

Acute pelvic inflammatory disease with tubo-ovarian abscess accounted for 3/46 (6.5%) and was managed by drainage and adhesiolysis with escalation to definitive surgery as indicated; where performed, the operative time for TOA management with adhesiolysis/partial oophorectomy was 120 minutes (100-150), within the spectrum reported in multi-centre series by Reich and McGlynn [11].

Endometriotic cysts (2/46; 4.3%) underwent deroofing with ovarian reconstruction (80 minutes; 60-100), while a separate case of endometriotic cyst rupture with bowel involvement required extensive adhesiolysis (160 minutes; 150-170); these timings align with procedure-matched reports and the fertility-preserving operative principles articulated in the RCOG Green-top Guideline No. 21 [17]. A single corpus luteal cyst rupture (1/46; 2.2%) was treated by cystectomy with bilateral oophoropexy, consistent with minimally invasive haemostatic strategies reported in contemporary cohorts (e.g., Kim JH et al. [18]).

Operative duration across the cohort tracked procedural complexity: 50 minutes (40-65) for isolated oophoropexy, 60 minutes (45-75) for cystectomy with oophoropexy, 70 minutes (50-90) for salpingectomy in ectopic pregnancy, and 80-160 minutes for advanced adhesiolysis or combined procedures (TOA management 120 minutes, endometriotic cyst deroofing 80 minutes, endometriotic cyst rupture with extensive adhesiolysis 160 minutes, and TLH + BSO with uterosacral colpopexy 120 minutes). These durations are comparable to reports in ectopic surgery, torsion, and complex inflammatory disease (Hajenius et al. [4]; Bar-On et al. [8]; Reich and McGlynn [11]), with prolongation in dense adhesiolysis paralleling the experience of Henry-Suchet et al. [12]. All procedures were completed laparoscopically with 0% conversion, favourably contrasting with historical conversion rates reported in broader gynaecological series (Mallick and Odejinmi [19]).

Complications occurred in 6/46 (13.0%); 40/46 (87.0%) had none. Event-wise, there were four episodes of low-grade fever, three perioperative transfusions, two instances of 24-hour HDU observation, and one transient postoperative ileus across these six patients. There were no readmissions and no re-laparoscopies. This safety profile aligns with contemporary laparoscopic torsion and adnexal-emergency series reporting low morbidity and very low conversion (Bar-On et al. [8]; Teng et al. [9]), and compares favourably with broader institutional audits (Kruszka and Kruszka [1]).

In summary, in haemodynamically stable patients, a laparoscopy-first strategy is feasible and effective across major emergency pathologies, achieving high completion without conversion, procedure-appropriate efficiency, and low perioperative morbidity-conclusions concordant with outcomes reported by Kruszka and Kruszka [1], Oelsner and Shashar [6], Alkatout et al. [3], and Hajenius et al. [4].

Limitations

Several limitations of this study must be acknowledged. First, the sample size of 46 patients drawn from a single tertiary care center is relatively small, which may limit the generalizability of the findings to broader or less specialized healthcare settings. Second, the prospective observational design inherently lacks a control or comparison group-such as patients managed via laparotomy or conservative approaches-which restricts the ability to perform direct comparative outcome analyses. Furthermore, the statistical analysis was purely descriptive by design, without formal hypothesis testing or subgroup comparisons. Finally, while the study demonstrates excellent early postoperative recovery, it lacks long-term follow-up data; assessing subsequent clinical endpoints such as future fertility rates, recurrence of adnexal torsion, and incidence of chronic pelvic pain remains necessary to fully evaluate the long-term benefits of this laparoscopy-first approach.

## Conclusions

Laparoscopy is a safe and effective diagnostic and therapeutic approach for the management of gynaecological emergencies in haemodynamically stable patients. It enables accurate diagnosis, facilitates timely pathology-directed treatment, and supports fertility- and organ-preserving management whenever clinically appropriate. When performed by experienced surgeons in appropriately selected patients, a laparoscopy-first approach can minimize perioperative morbidity while providing the benefits of minimally invasive surgery. These findings reinforce the role of laparoscopy as the preferred surgical modality for the management of gynaecological emergencies in tertiary care settings where the necessary expertise and resources are available.
